# Supplementary material for: The dominating mode of two competing massive modes of quadratic gravity
Source: Sci Rep. 2023 May 26;13:8547. doi: 10.1038/s41598-023-34802-8 (PMC10220037; doi:10.1038/s41598-023-34802-8)
Supplement: Supplementary file 1 — Supplementary Information. [file 41598_2023_34802_MOESM1_ESM.pdf]

Supplementary Material: Two competing massive modes in quadratic gravity: Which one dominates?

Avijit Chowdhury[1,*], Semin Xavier[1,+], and S. Shankaranarayanan[1,†]

[1]Department of Physics, Indian Institute of Technology Bombay, Mumbai 400076, India
[*]avijit.phy@iitb.ac.in
[+]seminxavier@iitb.ac.in
[†]shanki@phy.iitb.ac.in

ABSTRACT

This document contains details related to the calculations presented in the main text[1]. The equation numbers continues from the main text.

I Quadratic gravity as GR “minus" Massive Gravity

As first pointed out by Stelle[2], a generic quadratic theory of gravity is equivalent to GR “minus" massive gravity at a linear level in flat space. In this section, we will demonstrate an extension of the analysis to a Ricci-flat spacetime and prove the consistency of the decomposition presented in Eqs. (15) and (9). The generic quadratic gravity action in four dimensions is given by,

$$S_{\rm QG} = \frac{1}{2\kappa^2}\int d^4x\sqrt{-g}\left[R - 2\kappa^2\alpha R_{\mu\nu}R^{\mu\nu} + 2\kappa^2\beta R^2\right]. \tag{39}$$

Perturbing the action in (39) about the background metric ($\bar{g}_{\mu\nu}$) to second order in $h_{\mu\nu} = g_{\mu\nu} - \bar{g}_{\mu\nu}$, we get,

$$S^{(2)}_{QG}[h_{\mu\nu}] = \frac{1}{2\kappa^2}\int d^4x\sqrt{-\bar{g}}\Big[-h^{\mu\nu}\delta R_{\mu\nu}[h_{\mu\nu}] - 2\alpha\kappa^2\delta R_{\mu\nu}[h_{\mu\nu}]\delta R^{\mu\nu}[h_{\mu\nu}] + 2\beta\kappa^2(\delta R[h_{\mu\nu}])^2 + \frac{h}{2}\bar{g}^{\mu\nu}\delta R_{\mu\nu}\Big] \tag{40}$$

where, $h = \bar{g}^{\mu\nu}h_{\mu\nu}$. We now proceed by treating $\delta R_{\mu\nu}$ as an independent variable. However, this introduces extra degrees of freedom. In order to restrict the extra degrees of freedom, we replace $\delta R_{\mu\nu}$ in the above expression with an auxiliary variable $A_{\mu\nu}$, thus leading to:

$$S^{(2)}_{QG}[-h_{\mu\nu}, A_{\mu\nu}, \lambda_{\mu\nu}] = \frac{1}{2\kappa^2}\int d^4x\sqrt{-\bar{g}}\Big[-h^{\mu\nu}A_{\mu\nu} - 2\alpha\kappa^2 A_{\mu\nu}A^{\mu\nu} + 2\beta\kappa^2 A^2 + \frac{h}{2}\bar{g}^{\mu\nu}A_{\mu\nu} + \lambda^{\mu\nu}(A_{\mu\nu} - \delta R_{\mu\nu}[h_{\mu\nu}])\Big] \tag{41}$$

where $A = \bar{g}^{\mu\nu}A_{\mu\nu}$ and $\lambda_{\mu\nu}$ is the lagrange multiplier. Varying the above action with respect to $A_{\mu\nu}$, we get:

$$\lambda_{\mu\nu} = 4\alpha\kappa^2 A_{\mu\nu} - 4\beta\kappa^2\bar{g}_{\mu\nu}A + h_{\mu\nu} - \frac{1}{2}h\bar{g}_{\mu\nu}\ . \tag{42}$$

Rewriting the above expression in terms of $\lambda_{\mu\nu}$ and $h_{\mu\nu}$ and substituting the resultant in Eq. (41), we get:

$$S^{(2)}_{QG}[\Phi_{\mu\nu}, \tilde{\Phi}_{\mu\nu}] = \frac{1}{2\kappa^2}\int d^4x\sqrt{-\bar{g}}\Big[-\Phi^{\mu\nu}\delta R_{\mu\nu}\left[\Phi_{\mu\nu}\right] + \tilde{\phi}^{\mu\nu}\delta R_{\mu\nu}\left[\tilde{\phi}_{\mu\nu}\right] + \frac{m^2}{2}\left(\tilde{\Phi}_{\mu\nu}\tilde{\Phi}^{\mu\nu} - (1-\varepsilon)\tilde{\Phi}^2\right)\Big], \tag{43}$$

where

$$\lambda_{\mu\nu} = \Phi_{\mu\nu} - \tilde{\Phi}_{\mu\nu}; \qquad \tilde{h}_{\mu\nu} = h_{\mu\nu} - \frac{1}{2}h\bar{g}_{\mu\nu} = \Phi_{\mu\nu} + \tilde{\Phi}_{\mu\nu} \tag{44}$$

$$\varepsilon = \frac{1+\beta}{(\alpha - 4\beta)}; \qquad m^2 = \frac{1}{(\alpha\kappa^2)} \tag{45}$$

In the case of GUP-inspired Quadratic gravity, Eq. (42) reduces to:

$$h_{\mu\nu} = \psi_{\mu\nu} - \frac{1}{2}\bar{g}_{\mu\nu}\psi - 4\gamma\hat{A}_{\mu\nu} - \gamma A\bar{g}_{\mu\nu} \tag{46}$$

where, $\hat{A}_{\mu\nu} = A_{\mu\nu} - \frac{1}{4}\bar{g}_{\mu\nu}A$ is the traceless part of $A_{\mu\nu}$. Mapping $\lambda_{\mu\nu} \to \psi_{\mu\nu}$, $\hat{A}_{\mu\nu} \to \hat{R}^{(1)}_{\mu\nu}$ and $A \to R^{(1)}$, leads to Eqs. (15,9). The above analysis establishes the metric decomposition into massless spin-2, massive spin-0, and massive spin-2 modes. Combining the results of Section (II), this shows the equations of motion (14,16, 17) lead to the correct equations of motion.

It is possible to redo the above analysis for an arbitrary Einsteinian manifold, interms of the Einstein tensor instead of the Ricci tensor[3]. We represent the quadratic gravity action in Eq. (2) as:

$$S_{\rm QG} = \frac{1}{2\kappa^2}\int d^4x\sqrt{-g}\left[-G - 2\kappa^2\alpha G_{\mu\nu}G^{\mu\nu} + 2\kappa^2\beta G^2\right]. \tag{47}$$

Perturbing the action (47) about the background metric ($\bar{g}_{\mu\nu}$) to second order in $h_{\mu\nu} = g_{\mu\nu} - \bar{g}_{\mu\nu}$, we get,

$$S^{(2)}_{QG}[h_{\mu\nu}] = \frac{1}{2\kappa^2}\int d^4x\sqrt{-\bar{g}}\Big[h^{\mu\nu}\delta G_{\mu\nu}[h_{\mu\nu}] - 2\alpha\kappa^2\delta G_{\mu\nu}[h_{\mu\nu}]\delta G^{\mu\nu}[h_{\mu\nu}] + 2\beta\kappa^2(\delta G[h_{\mu\nu}])^2 - \frac{h}{2}\bar{g}^{\mu\nu}\delta G_{\mu\nu}\Big] \tag{48}$$

where, $h = \bar{g}^{\mu\nu}h_{\mu\nu}$. Replacing $\delta G_{\mu\nu}$ in the above expression by an auxiliary variable $A_{\mu\nu}$ and imposing a constraint, we get,

$$S^{(2)}_{QG}[h_{\mu\nu}, A_{\mu\nu}, \lambda_{\mu\nu}] = \frac{1}{2\kappa^2}\int d^4x\sqrt{-\bar{g}}\Big[-h^{\mu\nu}A_{\mu\nu} - 2\alpha\kappa^2 A_{\mu\nu}A^{\mu\nu} + 2\beta\kappa^2 A^2 - \frac{h}{2}\bar{g}^{\mu\nu}A_{\mu\nu} + \lambda^{\mu\nu}(A_{\mu\nu} - \delta G_{\mu\nu}[h_{\mu\nu}])\Big] \tag{49}$$

where $A = \bar{g}^{\mu\nu}A_{\mu\nu}$ and $\lambda_{\mu\nu}$ is the lagrange multiplier. Varying the above action with respect to $A_{\mu\nu}$, we get

$$\lambda_{\mu\nu} = 4\alpha\kappa^2 A_{\mu\nu} - 4\beta\kappa^2\bar{g}_{\mu\nu}A - h_{\mu\nu} + \frac{1}{2}h\bar{g}_{\mu\nu}\,. \tag{50}$$

Substituting $A_{\mu\nu}$ in terms of $\lambda_{\mu\nu}$ and $h_{\mu\nu}$ from Eq. (50) in Eq. (49) and using

$$\tilde{h}_{\mu\nu} = h_{\mu\nu} - \frac{1}{2}h\bar{g}_{\mu\nu} = \Phi_{\mu\nu} + \tilde{\Phi}_{\mu\nu}\,; \quad \lambda_{\mu\nu} = \tilde{\Phi}_{\mu\nu} - \Phi_{\mu\nu}, \tag{51}$$

we get,

$$S^{(2)}_{QG}[\Phi_{\mu\nu}, \tilde{\Phi}_{\mu\nu}] = \frac{1}{2\kappa^2}\int d^4x\sqrt{-\bar{g}}\Big[\Phi^{\mu\nu}\delta G_{\mu\nu}\left[\Phi_{\mu\nu}\right] - \tilde{\Phi}^{\mu\nu}\delta G_{\mu\nu}\left[\tilde{\Phi}_{\mu\nu}\right] + \frac{m^2}{2}\left(\tilde{\phi}_{\mu\nu}\tilde{\Phi}^{\mu\nu} - (1-\varepsilon)\tilde{\Phi}^2\right)\Big], \tag{52}$$

where, $\varepsilon = 1 + \beta/(\alpha - 4\beta)$, $m^2 = 1/(\alpha\kappa^2)$. For the GUP-inspired Stelle gravity, we once again recover the ansatz (15) with the following mapping $\psi_{\mu\nu} \to -\lambda_{\mu\nu}$, $\hat{R}^{(1)}_{\mu\nu} \to -\hat{A}_{\mu\nu}$ and $R^{(1)} \to -A$.

II Reduced equations of motion in Ricci-flat space-times

The field equation derived from the action (2) with arbitrary values of the parameters α and β is given by

$$R_{\mu\nu} - \frac{1}{2}Rg_{\mu\nu} + 4\beta R_{\mu\nu}R - 4\alpha R^{\rho\sigma}R_{\mu\rho\nu\sigma} + 2(\alpha - 2\beta)\nabla_\mu\nabla_\nu R - 2\alpha\Box R_{\mu\nu} + g_{\mu\nu}\left(\alpha R_{\rho\sigma}R^{\rho\sigma} + \beta R^2 + (\alpha - 4\beta)\Box R\right) = 0\,. \tag{53}$$

Linearizing the above field equation in a Ricci-flat background one gets

$$\delta R_{\mu\nu} - \frac{1}{2}\bar{g}_{\mu\nu}\delta R - 4\alpha\bar{R}_{\mu\rho\nu\sigma}\delta R^{\rho\sigma} + 2(\alpha - 2\beta)\bar{\nabla}_\mu\bar{\nabla}_\nu\delta R - 2\alpha\bar{\Box}\delta R_{\mu\nu} - \bar{g}_{\mu\nu}(\alpha - 4\beta)\bar{\Box}\delta R = 0\,. \tag{54}$$

Equation (54) can be written in terms of the metric perturbations[4] by expanding $\delta R_{\mu\nu}$ and δR in terms of $h_{\mu\nu}$ (see Eqs. (6,7)). We choose the following ansatz for the metric perturbations in Ricci-flat spacetime:

$$h_{\mu\nu} = \left[\psi_{\mu\nu} - \frac{\bar{g}_{\mu\nu}}{2}\psi\right] + \left[C_1 + \frac{C_2}{4}\right]R^{(1)}\bar{g}_{\mu\nu} - C_2\hat{R}^{(1)}_{\mu\nu}\,, \tag{55}$$

where C_1, C_2 are constants to be determined and $\psi_{\mu\nu}$ satisfies the transverse-traceless condition $\left(\bar{\nabla}^\mu\psi_{\mu\nu} = 0,\ \bar{g}^{\mu\nu}\psi_{\mu\nu} = 0\right)$. We demand that $R^{(1)}$ and $\hat{R}^{(1)}_{\mu\nu}$ to be respectively equal to the perturbed Ricci scalar δR and the *traceless part* of the perturbed Ricci tensor ($\delta R_{\mu\nu} - \frac{1}{4}\bar{g}_{\mu\nu}\delta R$). This will ensure that the linearized Bianchi identity (19) is satisfied at all times and in all spaces. Hence, equating δR with $R^{(1)}$ and comparing it with the trace of linearized field equation (54) we get,

$$C_2 = -3C_1 + 4(\alpha - 3\beta)\,. \tag{56}$$

Thus, we get the propagation equation for the massive spin-0 mode as,

$$\bar{\Box}R^{(1)} - R^{(1)}/4(3\beta-\alpha) = 0\,. \tag{57}$$

Similarly, equating $R^{(1)}_{\mu\nu} = \hat{R}^{(1)}_{\mu\nu} + \frac{1}{4}\bar{g}_{\mu\nu}R^{(1)}$ with $\delta R_{\mu\nu}$ and using Eq. (56) we get,

$$\begin{aligned}&\frac{1}{2}\Big[\Big(\bar{\Box}\psi_{\mu\nu} + 2\bar{R}_{\mu\rho\nu\sigma}\psi^{\rho\sigma}\Big) - (C_1 - 4\alpha + 12\beta)\Big(\bar{\nabla}_\mu\bar{\nabla}_\nu R^{(1)} - \frac{\bar{g}_{\mu\nu}R^{(1)}}{8(\alpha-3\beta)}\Big)\\&+(3C_1 - 4\alpha + 12\beta)\Big(\bar{\Box}R^{(1)}_{\mu\nu} + 2\bar{R}_{\mu\rho\nu\sigma}R^{(1)\rho\sigma}\Big)\Big] + R^{(1)}_{\mu\nu} = 0\end{aligned} \tag{58}$$

Comparing with Eq. (54) and using Eq. (8) we get,

$$\bar{\Box}\psi_{\mu\nu} + 2\bar{R}_{\mu\alpha\nu\beta}\psi^{\alpha\beta} = 0\,, \tag{59}$$

$$C_1 = -4\beta \text{ and } \alpha = 2\beta\,. \tag{60}$$

Equation (59) gives the dynamics of the massless spin-2 graviton mode, as $\psi_{\mu\nu}$ satisfies the traceless-transverse condition. Equation (60), on the other hand, implies that the decomposition scheme in Eq. (55) is specific to the GUP-inspired Stelle gravity ($\alpha = 2\beta = \gamma$), in which case $C_1 = -4\beta = -2\gamma$ and $C_2 = 4\gamma$. Thus, substituting C_1 and C_2 in Eq. (58) and taking cognizance of Eqs. (57), (59), (19) and (18), we get the propagation equation of the massive spin-2 mode as given in Eq. (17)

$$\bar{\Box}\hat{R}^{(1)}_{\mu\nu} + 2\bar{R}_{\mu\alpha\nu\beta}\hat{R}^{(1)\alpha\beta} - \hat{R}^{(1)}_{\mu\nu}/(2\gamma) = 0\,. \tag{61}$$

In the case of $\alpha = 2\beta = \gamma$, Eq. (57) reduces to Eq. (14). It is important to note that in the massless limit $\gamma \to 0$, Eqs. (14) and (17) suggest that $\hat{R}^{(1)}_{\mu\nu}$ and $R^{(1)}$ vanishes identically and the theory is described by only one massless spin-2 graviton $\psi_{\mu\nu}$.

III Coefficients in the effective GW stress-energy tensor

Using xAct Mathematica packages, $\mathscr{A}_{\mu\nu}, \mathscr{B}_{\mu\nu}, \mathscr{C}_{\mu\nu}$, and $\mathscr{D}_{\mu\nu}$ in Eq. (22) are obtained as:

$$\mathscr{A}_{\mu\nu} = -\bar{R}_{\mu\lambda\alpha\rho}\psi^\alpha_\nu\psi^{\lambda\rho} - \frac{1}{4}\bar{g}_{\mu\nu}\bar{R}_{\alpha\rho\lambda\sigma}\,\psi^{\alpha\lambda}\psi^{\rho\sigma} \tag{62}$$

$$\begin{aligned}\mathscr{B}_{\mu\nu} =\;& 2\bar{R}_{\nu\ \alpha}^{\ \sigma\ \eta}\bar{R}_{\lambda\sigma\rho\eta}\psi^\alpha_\mu\psi^{\lambda\rho} - 4\bar{R}_{\mu\alpha\lambda}{}^{\eta}\bar{R}_{\nu\rho\sigma\eta}\psi^{\alpha\lambda}\psi^{\rho\sigma} - 2\bar{R}_{\mu\alpha\nu}{}^{\eta}\bar{R}_{\lambda\rho\sigma\eta}\psi^{\alpha\lambda}\psi^{\rho\sigma} - \bar{R}_{\mu\rho\nu\sigma}\bar{\nabla}^\rho\psi^{\alpha\lambda}\bar{\nabla}^\sigma\psi_{\alpha\lambda}\\&+4\bar{R}_\mu{}^\eta{}_{\nu\alpha}\bar{R}_{\lambda\rho\sigma\eta}\psi^{\alpha\lambda}\psi^{\rho\sigma} - \bar{g}_{\mu\nu}\bar{R}_\alpha{}^\eta{}_\lambda{}^h\bar{R}_{\rho\eta\sigma h}\psi^{\alpha\lambda}\psi^{\rho\sigma} + 2\bar{R}_{\alpha\rho\lambda\sigma}\bar{\nabla}_\mu\psi^{\alpha\lambda}\bar{\nabla}_\nu\psi^{\rho\sigma} - 2\bar{R}_{\alpha\rho\lambda\sigma}\bar{\nabla}_\mu\psi^{\rho\sigma}\bar{\nabla}^\lambda\psi_\nu{}^\alpha\\&+2\bar{R}_{\alpha\lambda\rho\sigma}\bar{\nabla}_\nu\psi_\mu{}^\alpha\bar{\nabla}^\sigma\psi^{\lambda\rho} - 2\bar{R}_{\alpha\lambda\rho\sigma}\bar{\nabla}^\alpha\psi_{\mu\nu}\bar{\nabla}^\sigma\psi^{\lambda\rho}\end{aligned} \tag{63}$$

$$\begin{aligned}\mathscr{C}_{\mu\nu} =\;& 8\bar{R}_{\nu\lambda\alpha\rho}\hat{R}^{(1)}{}_\mu{}^\alpha\hat{R}^{(1)\lambda\rho} + 2\bar{R}_{\mu\lambda\nu\rho}(4\hat{R}^{(1)}{}_\alpha{}^\rho\hat{R}^{(1)\alpha\lambda} - 7R^{(1)}\hat{R}^{(1)\lambda\rho}) - 8\bar{R}_{\alpha\lambda\rho\sigma}\bar{\nabla}_\nu\hat{R}^{(1)}{}_\mu{}^\alpha\bar{\nabla}^\sigma\psi^{\lambda\rho}\\&+4\bar{g}_{\mu\nu}R_{\alpha\rho\lambda\sigma}\hat{R}^{(1)\alpha\lambda}\hat{R}^{(1)\rho\sigma} - 16\bar{R}_{\alpha\rho\lambda\sigma}\bar{\nabla}_\mu\psi^{\rho\sigma}\bar{\nabla}_\nu\hat{R}^{(1)\alpha\lambda} + 2\bar{R}_{\nu\rho\lambda\sigma}\bar{\nabla}_\mu\psi^{\rho\sigma}\bar{\nabla}^\lambda R^{(1)}\\&+8\bar{R}_{\alpha\rho\lambda\sigma}\bar{\nabla}_\mu\psi^{\rho\sigma}\bar{\nabla}^\lambda\hat{R}^{(1)}{}_\nu{}^\alpha + 2\bar{R}_{\nu\lambda\rho\sigma}\bar{\nabla}^\lambda R^{(1)}\bar{\nabla}^\sigma\psi_\mu{}^\rho + 2\bar{R}_{\nu\sigma\lambda\rho}\bar{\nabla}^\lambda R^{(1)}\bar{\nabla}^\sigma\psi_\mu{}^\rho\\&+4\bar{R}_{\mu\rho\nu\sigma}\bar{\nabla}^\rho\hat{R}^{(1)\alpha\lambda}\bar{\nabla}^\sigma\psi_{\alpha\lambda} + 4\bar{R}_{\mu\sigma\nu\rho}\bar{\nabla}^\rho\hat{R}^{(1)\alpha\lambda}\bar{\nabla}^\sigma\psi_{\alpha\lambda} - 10\bar{R}_{\mu\lambda\rho\sigma}\bar{\nabla}_\nu R^{(1)}\bar{\nabla}^\sigma\psi^{\lambda\rho}\\&+8\bar{R}_{\alpha\lambda\rho\sigma}\bar{\nabla}^\alpha\hat{R}^{(1)}{}_{\mu\nu}\bar{\nabla}^\sigma\psi^{\lambda\rho} + 2\bar{g}_{\mu\nu}\bar{R}_{\lambda\rho\sigma\eta}\bar{\nabla}^\lambda R^{(1)}\bar{\nabla}^\eta\psi^{\rho\sigma}\end{aligned} \tag{64}$$

$$\begin{aligned}\mathscr{D}_{\mu\nu} = 4\Big(&8\bar{R}_\nu{}^\sigma{}_\alpha{}^\eta\bar{R}_{\lambda\sigma\rho\eta}\hat{R}^{(1)}{}_\mu{}^\alpha\hat{R}^{(1)\lambda\rho} - 16\bar{R}_{\mu\alpha\lambda}{}^\eta\bar{R}_{\nu\rho\sigma\eta}\hat{R}^{(1)\alpha\lambda}\hat{R}^{(1)\rho\sigma} + 2\bar{R}_{\nu\sigma\lambda\rho}\bar{\nabla}^\lambda R^{(1)}\bar{\nabla}^\sigma\hat{R}^{(1)}{}_\mu{}^\rho\\&-8\bar{R}_{\mu\alpha\nu}{}^\eta\bar{R}_{\lambda\rho\sigma\eta}\hat{R}^{(1)\alpha\lambda}\hat{R}^{(1)\rho\sigma} + 16\bar{R}_\mu{}^\eta{}_{\nu\alpha}\bar{R}_{\lambda\rho\sigma\eta}\hat{R}^{(1)\alpha\lambda}\hat{R}^{(1)\rho\sigma}\\&-4\bar{g}_{\mu\nu}\bar{R}_\alpha{}^\eta{}_\lambda{}^h\bar{R}_{\rho\eta\sigma h}\hat{R}^{(1)\alpha\lambda}\hat{R}^{(1)\rho\sigma} + 8\bar{R}_{\alpha\rho\lambda\sigma}\bar{\nabla}_\mu\hat{R}^{(1)\alpha\lambda}\bar{\nabla}_\nu\hat{R}^{(1)\rho\sigma}\\&+\bar{R}_{\mu\lambda\nu\sigma}\bar{\nabla}^\lambda R^{(1)}\bar{\nabla}^\sigma R^{(1)} + 2\bar{R}_{\nu\lambda\rho\sigma}\bar{\nabla}^\lambda R^{(1)}\bar{\nabla}^\sigma\hat{R}^{(1)}{}_\mu{}^\rho - 4\bar{R}_{\mu\rho\nu\sigma}\bar{\nabla}^\rho\hat{R}^{(1)\alpha\lambda}\bar{\nabla}^\sigma\hat{R}^{(1)}{}_{\alpha\lambda}\Big)\end{aligned} \tag{65}$$

As mentioned earlier, we assume $\psi(V,\rho)$ and $P(V,\rho)$ to be slowly varying close to the event horizon ($\rho \sim 2M_0$). The leading order solid angle-averaged value (of the $\rho - V$ component) of the effective GW stress-energy tensor (up to leading order

corrections in γ) used in Eq. (37) in the Schwarzschild background is given by

$$
\begin{aligned}
t_V^{\rho\mathrm{GW}} \approx & \frac{M_0^4\psi'(V,\rho)}{21\pi}\left\{8[21o^{00}\iota^{22}+20o^{23}\iota^{23}+8o^{33}\iota^{33}]\gamma P'(V,\rho)+[21(o^{22})^2+20(o^{23})^2+8(o^{33})^2]\psi'(V,\rho)\right\} \\
& +\frac{1}{21\pi}M_0^2\psi'(V,\rho)\left[-4(21o^{12}\iota^{02}+10o^{13}\iota^{03}+21o^{02}\iota^{12}+10o^{03}\iota^{13})\gamma P'(V,\rho)+\right. \\
& \left.\left(21o^{02}o^{12}+10o^{03}o^{13}+\gamma\left(21(o^{22})^2-20(o^{23})^2+40o^{22}\iota^{33}+8(o^{33})^2\right)\right)\psi'(V,\rho)\right] \\
& +\frac{1}{21\pi}\left(21o^{12}\iota^{02}+10o^{13}\iota^{03}-21o^{02}\iota^{12}-10o^{03}\iota^{13}\right)M_0\gamma\left[\psi(V,\rho)\mathrm{P}'(V,\rho)-\mathrm{P}(V,\rho)\psi'(V,\rho)\right] \\
& +\frac{1}{672M_0^2\pi}\left[84(o^{11}\iota^{00}+o^{00}\iota^{11})\gamma P(V,\rho)\psi(V,\rho)+\left(-21o^{00}o^{11}+2\left(-42o^{02}o^{12}+21(o^{12})^2\right.\right.\right. \\
& \left.\left.\left.-20o^{03}o^{13}+10(o^{13})^2-63o^{11}o^{22}+38o^{11}o^{33}\right)\gamma\right)\left(\psi(V,\rho)\right)^2+84(o^{01})^2\gamma(\psi'(V,\rho))^2\right] \\
& -\frac{1}{32M_0^3\pi}\left(2(o^{01})^2+o^{00}o^{11}-2o^{01}o^{11}\right)\gamma\psi(V,\rho)\psi'(V,\rho)+\frac{1}{64M_0^4\pi}(\psi(V,\rho))^2\gamma\left[-2(o^{01})^2+o^{00}o^{11}\right] \\
& +\frac{\gamma}{168\pi}\left[42(-o^{11}\iota^{00}+o^{00}\iota^{11})P(V,\rho)\psi'(V,\rho)+\psi(V,\rho)\left(42(o^{11}\iota^{00}-o^{00}\iota^{11})P'(V,\rho)+\left(21o^{02}o^{12}\right.\right.\right. \\
& \left.\left.\left.-21(o^{12})^2+10o^{03}o^{13}-10(o^{13})^2-42o^{11}o^{22}-20o^{11}o^{33}\right)\psi'(V,\rho)\right)\right] \\
& +\frac{1}{336\pi}\left[4(21o^{12}\iota^{02}+10o^{13}\iota^{03}+21o^{02}\iota^{12}+10o^{03}\iota^{13})\gamma P(V,\rho)\psi(V,\rho)\right. \\
& -\left(21o^{02}o^{12}+42(o^{12})^2+10o^{03}o^{13}+20(o^{13})^2-42o^{11}o^{22}-20o^{11}\iota^{33}\right)\psi(V,\rho)^2 \\
& +2\psi'(V,\rho)\left\{-84(o^{11}\iota^{00}+2o^{01}\iota^{01}+o^{00}\iota^{11})\gamma P'(V,\rho)+\left(21(o^{01})^2\right.\right. \\
& \left.\left.\left.+21o^{00}o^{11}+252o^{02}o^{12}\gamma+120o^{03}o^{13}\gamma-84o^{01}o^{22}\gamma-40o^{01}\iota^{33}\gamma\right)\psi'(V,\rho)\right\}\right]
\end{aligned}
\tag{66}
$$

where $'$ denotes derivative wrt V.

References

1. Chowdhury, A., Xavier, S. & Shankaranarayanan, S. Two competing massive modes in quadratic gravity: Which one dominates? (2023).
2. Stelle, K. S. Classical Gravity with Higher Derivatives. *Gen. Rel. Grav.* **9**, 353–371, DOI: 10.1007/BF00760427 (1978).
3. Tachinami, T., Tonosaki, S. & Sendouda, Y. Gravitational-wave polarizations in generic linear massive gravity and generic higher-curvature gravity. *Phys. Rev. D* **103**, DOI: 10.1103/physrevd.103.104037 (2021).
4. Misner, C. W., Thorne, K. S. & Wheeler, J. A. *Gravitation* (W. H. Freeman, San Francisco, 1973).
